# The protective role of the microenvironment in hairy cell leukemia treatment: Facts and perspectives

**DOI:** 10.3389/fonc.2023.1122699

**Published:** 2023-03-08

**Authors:** Ernesto Gargiulo, Mirta Giordano, Carsten U. Niemann, Etienne Moussay, Jérôme Paggetti, Pablo Elías Morande

**Affiliations:** ^1^ Tumor Stroma Interactions – Department of Cancer Research, Luxembourg Institute of Health Luxembourg, Luxembourg; ^2^ Chronic Lymphocytic Leukemia Laboratory, Department of Hematology, Rigshospitalet, Copenhagen, Denmark; ^3^ PERSIMUNE, Department of Infectious Diseases, Rigshospitalet, Copenhagen, Denmark; ^4^ Instituto de Medicina Experimental (IMEX)-CONICET, Academia Nacional de Medicina, Buenos Aires, Argentina; ^5^ Department of Clinical Medicine, University of Copenhagen, Copenhagen, Denmark

**Keywords:** HCL, leukemia microenvironment, treatment resistance, microenvironment targeting, novel therapies

## Abstract

Hairy cell leukemia (HCL) is an incurable, rare lymphoproliferative hematological malignancy of mature B cAlthough first line therapy with purine analogues leads to positive results, almost half of HCL patients relapse after 5-10 years, and standard treatment may not be an option due to intolerance or refractoriness. Proliferation and survival of HCL cells is regulated by surrounding accessory cells and soluble signals present in the tumor microenvironment, which actively contributes to disease progression. *In vitro* studies show that different therapeutic approaches tested in HCL impact the tumor microenvironment, and that this milieu offers a protection affecting treatment efficacy. Herein we explore the effects of the tumor microenvironment to different approved and experimental therapeutic options for HCL. Dissecting the complex interactions between leukemia cells and their milieu will be essential to develop new targeted therapies for HCL patients.

## 1 Introduction

### 1.1 Hairy cell leukemia

Representing approximately 2% of all leukemia cases worldwide, hairy cell leukemia (HCL) is an incurable lymphoproliferative B cell malignancy with an incidence rate of 0.3/100 000 in men and 0.1/100 000 in women (1, 2). The median age of HCL patients at diagnosis is close to 54 years (3, 4). The disease is characterized by the presence of abnormal B cells with hairy projections, which progressively accumulate in bone marrow (BM), spleen (causing splenomegaly) and other organs (e.g. liver) leading to a reduction of circulating erythrocytes, white blood cells, and platelets (known as pancytopenia) (5, 6). In contrast to chronic lymphocytic leukemia (CLL), HCL cells (HC) rarely infiltrate lymph nodes (3, 7). Patients affected by HCL can experience fatigue, increased risk of infections and bleeding due to anemia, leukopenia, and thrombocytopenia, respectively. Furthermore, HC infiltration in BM and other organs can lead to increased probability of fractures and impaired organ functions (3).

At diagnosis, HC are present at low frequency in peripheral blood (PB) and are characterized by the expression of typical B cell markers (like CD19, CD20, or CD22), as well as CD25, CD11c, CD103 and CD123, and by the mutation of the B-Raf proto-oncogene (BRAF, *BRAF^V600E^*) (8, 9). The latter, in particular, has been identified as a key mutation for the classic HCL subgroup (HCLc), while it is undetected in the variant form of HCL (HCLv) and in patients with IGHV4-34^+^. HCLv represents nearly 10% of all HCL cases, and up to 20% of patients belong to the IGHV4-34 molecular variant subgroup (10). BRAF^V600E^ mutation has been identified within the hematopoietic stem cell compartment, suggesting an early transformation stage leading to HCLc (11). HCLv (12) and IGHV4-34 (13) groups display a distinct molecular pathogenesis (14). Beyond BRAF^V600E^ mutation, HC express the anti-apoptotic B-cell lymphoma 2 (BCL-2) protein, a well-studied inhibitor of cell death that sustains cell survival, tumor growth and cancer disease progression (15–17). Standard treatment of HCL with cladribine (CDA) or pentostatin (2’-deoxycoformycin, DCF), alone or in combination with anti-CD20 (rituximab) immunotherapy, leads to remission in the vast majority of patients with certain subgroups of HCL patients can have a life expectation close to healthy individuals (18). However, no plateau on progression-free survival (PFS) curves has been achieved, thus most patients eventually relapse (19). Furthermore, the combined immune deficiencies due to HCL itself and to the treatments lead to high risk of infections during the first months after initiating therapy with CDA or DCF (20). This opens up to novel therapy strategies (21–24), clinical trials (detailed in **Table 1**), and basic research studies (25).

**Table 1:** Current clinical trials in HCL.

NCT CodeAA3:F25	Status	Clinical phases	Conditions	Interventions
NCT02131753	Recruiting	Phase 2|Phase 3	HCL	Cladribine
NCT05388123	Recruiting	Phase 2	HCL	Low dose vemurafenib and Rituximab
NCT04322383	Recruiting	Phase 2	HCL	Binimetinib
NCT03805932	Recruiting	Phase 1	HCL	Moxetumomab Pasudotox-tdfk, Rituximab and Ruxience
NCT04815356	Recruiting	Phase 1	c, v and r HCL	αCD22 CAR-T cells
NCT00923013	Recruiting	Phase 2	HCL	Cladribine and Rituximab
NCT01711632	Active, nr	Phase 2	HCL	Vemurafenib
NCT00321555	Active, nr	Phase 2	HCL	Anti-Tac(Fv)-PE38 (LMB-2) Immunotoxin
NCT01059786	Recruiting	Phase 2	HCL	Pentostatin, Rituximab and Bendamustine
NCT00412594	Recruiting	Phase 2	c and r HCL	Cladribine, rituximab and laboratory biomarker analysis
NCT01841723	Active, nr	Phase 2	c, v and r HCL	Ibrutinib
NCT03410875	Active, nr	Phase 2	HCL*	Vemurafenib and Obinutuzumab
NCT04324112	Recruiting	Phase 2	HCL	Binimetinib and Encorafenib
NCT04125290	Recruiting	Phase 3	Relapsed or refractory HCL	Moxetumomab Pasudotox-tdfk
NCT02560883	Recruiting	Not Applicable	HCL*	Clinical data collection
NCT05537766	Not yet recruiting	Phase 2	Relapsed/Refractory HCL*	Cyclophosphamide, Fludarabine and αCD19 CART cells
NCT01087333	Recruiting	Not Applicable	HCL*	Clinical sample collection
NCT04578600	Recruiting	Phase 1	r and refractory HCL*	Lenalidomide, Obinutuzumab and Azacitidine
NCT04681105	Recruiting	Phase 1	r and refractory HCL*	Acetaminophen, Dexamethasone, Diphenhydramine, Flotetuzumab, Ibuprofen and Ranitidine
NCT02362035	Active, nr	Phase 1|Phase 2	HCL*	Acalabrutinib and Pembrolizumab
NCT02213913	Active, nr	Phase 1|Phase 2	HCL and progressive HCL*	Lenalidomide, Etoposide, Prednisone, Vincristine sulfate, Doxorubicin Hydrochloride, Cyclophosphamide, Rituximab, quality-of-life assessment and laboratory biomarker analysis
NCT04952974	Recruiting		HCL*	Laboratory biomarker analysis
NCT04775745	Recruiting	Phase 1	HCL*	LP-168
NCT02153580	Active, nr	Phase 1	r HCL*	Bendamustine Hydrochloride, Cyclophosphamide, Etoposide, Fludarabine Phosphate and αCD19 CART cells
NCT01760655	Recruiting	Phase 2	HCL and refractory HCL*	Fludarabine Phosphate, Thiotepa, Cyclophosphamide, Tacrolimus, Mycophenolate mofetil, allogeneic lymphocytes, total body irradiation, HSCT and peripheral blood stem cell transplantation
NCT01815749	Active, nr	Phase 1	Post-transplant Refractory HCL*	αCD19 CAR-T cells, HSCT and laboratory biomarker analysis
NCT02924402	Recruiting	Phase 1	HCL*	XmAb13676
NCT01137643	Recruiting	Not Applicable	HCL*	Biologic sample preservation procedure and cytology specimen collection procedure
NCT01137825	Recruiting	Not Applicable	HCL*,**	Clinical data collection
NCT00935090	Recruiting	Not Applicable	HCL*,**	3’-deoxy-3’-[18F]fluorothymidine

nr, not recruiting; HCL, hairy cell leukemia; c, classic; v, variant; r, recurrent; HSCT, hematopoietic stem cell transplantation; CAR-T, Chimeric antigen receptor T; *, other hematologic malignancies; **, solid tumors.

### 1.2 Characteristics of the tumor microenvironment in HCL

Over the last decades, there has been an evident broadening in the research interests and in the design of treatment strategies from studying exclusively tumor cells, to also consider components of the surrounding microenvironment. This includes deep characterization of different cellular subsets and soluble components, as well as understanding the complex communicational network within the tumor microenvironment (TME) (26, 27). CLL represents one clear case of such shift to this new wider understanding and design in therapies (28).

In HCL, the BM represents a key anatomical site for the disease, where malignant cells proliferate and survive thanks to physical protection and constitutive signals provided from the different microenvironment cells. Sinusoidal endothelial cells, mesenchymal stromal cells (BMSCs) and osteoclasts, express high amounts of CXCL12. This allows hematopoietic stem cells (HSCs) to migrate from the endosteal to the vascular niche replenishing the pool of mature circulating blood cells (29). Given their high expression of CXCR4, HC are strongly attracted to the BM, as well as to the splenic and hepatic niches, where they physically interact with sinusoid cells expressing vascular cell adhesion molecule 1 (VCAM-1) (30). The absence of HC in lymph nodes is due to the lack of expression of the chemokine receptors CXCR5 and CCR7 (31). HC release tumor necrosis factor alpha (TNF-α), which stimulates VCAM-1 expression on surrounding endothelial cells, increasing tumor cell migration *in situ* (32). Beyond stimulating malignant B cell migration, BMSCs sustain HC survival and proliferation by interacting with the integrin α4β1 (very late antigen-4, VLA-4), expressed on malignant cells, triggering mitogen activated protein (MAP) kinases and the nuclear factor kappa-light-chain-enhancer of activated B cells (NF-κB) downstream pathway (33). To further sustain HC interaction with the extracellular matrix and sinusoidal endothelial cells in the microenvironment, tumor cells express CD44 that binds to hyaluronic acid, present in both BM and hepatic niches, and the integrin α_V_β_3_ binding the platelet/endothelial cell adhesion molecule 1 (PECAM-1) (34). Interactions between laminin and the basement membrane causes endothelial cell replacement by HC in the microenvironment (35). This represent a unique HCL vascular feature, taking place mainly in spleen (splenic pseudosinuses) and liver (hepatic hemangiomatous lesions) (36).

The T cell compartment is also altered in HCL (37). Thus, the expansion of T cells characterized by redundant T-cell receptor β variable region and high reactivity towards HC-surface CD40 results in a skewed T repertoire (38, 39). Given that CD40 downstream signals (MAPK and NF-κB pathways) are essential for HC proliferation (40), the expanded CD40L^+^ T cells in HCL are thought to have a tumor supportive function rather than being involved in disease suppression (37).

Furthermore, engagement of the B cell receptor (BCR) represents an important event during HCL pathogenesis. The vast majority of HCL patients show HC characterized by mutated immunoglobulin variable region genes (M-IGHV) (41, 42). The minor fraction of HCL cases with unmutated IGHV (UM-IGHV) display higher response to BCR stimulation compared with M-IGHV (43). Within the HCL microenvironment, BCR signaling could be potentially triggered by classical ligand interaction (e.g. auto-antigen) or through a ligand-independent fashion (tonic signaling). In both cases, BCR downstream signaling activates key kinases (SYK, BTK and PI3Kδ), leading to HC proliferation and survival. Moreover, BCR engagement also triggers the release of the chemokines CCL3 and CCL4, used to coordinate monocytes and T cell recruitment to the microenvironment (44–46).

## 2 Therapeutic options in HCL and the impact of the tumor microenvironment

### 2.1 Non-targeted agents for HCL: Cytokine alpha-interferon and purine analogs

**Figure 1:**
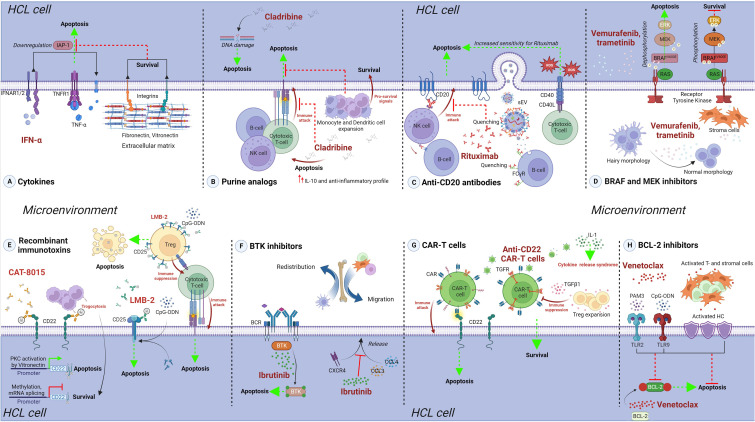
The protective role of the tumor microenvironment against treatments in HCL. Approved and experimental therapeutic options for HCL are presented, and details of microenvironment-mediated protection are provided. **(A)** Cytokines. IFN-α treatment efficacy is reduced due to engagement of HC to FN or VN receptors, present in the extracellular matrix of the spleen and bone marrow, inhibiting the IFN-α-mediated downregulation of IAPs, ultimately leading to reduced cell death. **(B)** Purine analogs. Cladribine off-targets effects could reduce the immune response against HC, mediated by cytotoxic T- and NK cells, and increase the levels of IL-10, promoting an anti-inflammatory profile. Indirect expansion of monocytes and dendritic cells can further favor HC survival. **(C)** Anti-CD20 antibodies. Monoclonal antibody Rituximab efficacy could be highly reduced in HCL due to the secretion of leukemia- and normal B cell-derived CD20^+^ sEV, as well as the higher expression of CD20 on normal B cells. On the other hand, CD40-CD40L interaction leads to increased sensitivity towards Rituximab in CLL and this could also be the case in HCL. **(D)** BRAF and MECK inhibitors. The pro-apoptotic effect of vemurafenib and trametinib is reduced by the presence of stromal cells in the microenvironment, which impair the dephosphorylation changes induced by these drugs. **(E)** Recombinant immunotoxins. The effect of these molecules could be affected by microenvironment modulation of the surface targets, e.g. CD22 availability during CAT-8015 treatment; or increased, e.g. CD25 upregulation during LMB-2 treatment in presence of CpG-ODN. Apoptosis of off-target cells, as regulatory T cells in the case of LMB-2, could indirectly influence the efficacy of the treatment. **(F)** BTK inhibitors. Ibrutinib treatment is highly efficient in affecting HC, but reduces the secretion of CCL3 and CCL4, and impairs CXCR4 signaling. This could possibly lead to redistribution of HC and other supporting cells in the microenvironment, possibly influencing other treatment regiments. **(G)** CAR-T cells. Anti-CD22 CAR-T therapy can be impaired by TGF-β1, directly affecting engineered T cells, as well as inducing Treg expansion. High levels of IL-1 in HCL microenvironment enhance the risk of CRS. **(H)** BCL-2 inhibitors. Venetoclax treatment efficacy is strongly reduced against HC stimulated with TLR2 and TLR9 ligands, as well as by the presence of activated T and stromal cells. Created with BioRender.com.

Before the significant improvement in cancer therapy that occurred with the advent of purine analogs, HCL was mainly treated either through splenectomy, chemotherapy with chlorambucil, rubidazone or methotrexate (among other drugs with more limited efficacy, reviewed in (46) and (47)), or with immune response modifiers such as interferons (47, 48). In 1984, Quesada et al. (49) suggested the use of the cytokine alpha-interferon (IFN-α) by intramuscular route and in 2002, Baker and colleagues deepened into its mechanisms of action (50). They showed that IFN-α exerts its cell death effect on HC by triggering autocrine production of TNF-α and mediating a suppression of inhibitor of apoptosis protein-1 (IAP-1) expression (**Figure 1A**). Importantly, engagement of the receptors for fibronectin (FN) or vitronectin (VN) in HC prevented this IFN-α-induced downregulation of IAPs, reducing its cytotoxicity. The high abundance of FN and VN in the extracellular matrix of HCL patient’s spleen and BM (51), together with the constitutive expression of integrins at the surface of HC binding these ligands (52), evidence a microenvironment-mediated protection towards IFN-α treatment.

The introduction of purine analogs implied a major change in the disease outcome of HCL. Indeed, first line treatment with CDA is still the initial option in the majority of cases to date (1), more than 30 years after its initial use. Alternatively, DCF was widely used with excellent results as well, but preference towards CDA became more frequent probably due to a shorter administration scheme (53). The *in vitro* effect of CDA on PB mononuclear cells of healthy donors shows a reduced proliferative capacity of T and B cells, but not NK cells (54). CDA also impairs the activation and increases the apoptosis of T, B and NK cells in a dose-dependent manner, negatively affects dendritic cells (55), and modulates the cytokine response towards an anti-inflammatory profile (56). Thus, despite being highly effective against HC, CDA also causes severe harm to HCL microenvironment cells such as CD56^+^ NK cells, CD8^+^ and CD4^+^ T cell subsets, and induces profound changes in the composition of soluble factors including an increase in interleukin (IL)-10 production, overall reducing immune surveillance and function (**Figure 1B**). This is clinically reflected by the high risk of infections in the first weeks and months after treatment of HCL. Interestingly, a recent report making use of BM trephine samples from HCL patients before and after CDA therapy showed a reduction in tumor infiltrating NK and T cells, while proportions of monocytes and dendritic cells increase (57). Since monocytes and macrophages have the capacity to induce HC proliferation by direct interaction *in vitro* (58), these over represented myeloid cells could play a key role in sustaining the survival of remnant leukemic cells after CDA therapy.

### 2.2 Immunotherapy with anti-CD20 antibodies

HCL cases that relapse before 2 years after initial therapy, as well as HCLv patients, are treated with CDA plus rituximab (1). As first line therapy, this combination has so far showed promising results, which further improve when administered in a sequential scheme (59). Rituximab is an anti-CD20 monoclonal antibody successfully used in CLL and different B lymphomas (60) that exerts its cell death effect in normal and neoplastic B cells by initiating the complement cascade and, mainly, through antibody dependent cellular cytotoxicity (ADCC) mediated by NK and monocyte/macrophages (61). Another anti-CD20 tested in HCL is obinutuzumab (62, 63), a second generation monoclonal antibody currently undergoing two different clinical trials (NCT04578600 and NCT03410875, **Table 1**). Obinutuzumab was used in combination with chlorambucil for treatment-naive CLL patients, showing superiority as compared to chlorambucil plus rituximab (64), and was also tested *in vitro*, where an improved ADCC towards CLL cells was detected when it was compared with rituximab (65). Another second generation anti-CD20 antibody used for refractory or intolerant CLL cases is ofatumumab, which binds a CD20 epitope different from the CD20 binding site of rituximab and obinutuzumab, that partially overlap (66). To the best of our knowledge, ofatumumab has not been tested in HCL yet.

In CLL, microenvironment-mediated stimulation of leukemia cells through CD40 leads to an increase in their sensitivity to rituximab (67). On the other side, CD20^+^ small extracellular vesicles (sEV) released in the tumor microenvironment by both CLL and normal B cell have the capacity to quench this antibody and decrease its availability for neoplastic cells (68). These or other possible effects linking anti-CD20 antibodies to the TME in HCL have not been studied so far. It is reported, however, that expression of CD20 is higher in normal B cells than in HC (69). Therefore, it is expected that normal B cells will be negatively affected by anti-CD20 based therapies, and that the CD20^+/hi^ sEV secreted by these cells will actively reduce their availability (**Figure 1C**). Additional effects that could be mediated by other leukocytes binding anti-CD20 antibodies through the Fc gamma Receptors (FcγR), involving mechanisms such as phagocytosis and cytokine release, remain to be elucidated in HCL.

### 2.3 Additional therapeutic options in HCL: BRAF and MEK inhibitors, immunotoxins, and BTK inhibitors

HCL patients from the classic group carry the BRAF^V600E^ mutation, while it is virtually absent in other B-cell leukemias and lymphomas (9, 70). Inhibiting BRAF-related signaling pathways represents a very interesting therapeutic approach that has been tested in the last decade in HCL, showing promising results (71). Pettirossi and colleagues showed that vemurafenib and trametinib, BRAF and MEK inhibitors respectively, cause strong MEK/ERK dephosphorylation and silence the transcriptional output of the activated BRAF-MEK-ERK pathway leading to the loss of the hairy morphology and to apoptosis (29). Treatment of relapsed or refractory HCL with vemurafenib as monotherapy leads to high overall response rates and 1-year PFS above 70% (72); while the combination of vemurafenib with trametinib has reported 89% overall response rates with 2-year PFS of 94% (73). Interestingly, stromal cells can partially protect HC from the cell death effect induced by BRAF inhibition (**Figure 1D**). Indeed, dephosphorylation of the BRAF-MEK-ERK pathway by the BRAF inhibitors vemurafenib and dabrafenib is reduced when HC are co-cultured with the stroma cell line HS-5, decreasing the drug’s pro-apoptotic effect (29). Thus, cells present in the HCL tumor microenvironment have the capacity to thwart dephosphorylation changes induced by certain drugs used in therapy, having a concrete impact on leukemic cells survival.

Recombinant immunotoxins are engineered chimeric proteins formed by a monoclonal antibody fragment fused to toxin, such as Pseudomonas exotoxin A (PE) (74). Once bound to its target by the antibody part, immunotoxins are internalized inducing cell death by arrest of protein synthesis (75). In HCL, the first immunotoxin tested, LMB-2, is directed against CD25 and showed a marked cytotoxicity against HC *in vitro* (76). In patients, LMB-2 showed positive results and achieved, in some cases, complete remission (77, 78). Importantly, regulatory T cells (Tregs), expressing CD25, have a key role in tumor immunosuppression in different lymphoid malignancies such as CLL, and represent a target for novel therapeutic approaches (79). LMB-2 eliminates human PB-derived Tregs *in vitro* (80), and selectively reduces circulating and tumor infiltrating Tregs in melanoma patients (81). Little is known about Tregs in HCL and there is no information about the effect of LMB-2 towards this cell subset in HCL. Still, these works open the question if LMB-2 could have also an impact on the Tregs present in the HCL-TME, which may indirectly contribute to the efficacy of treatment (**Figure 1E**). On the other hand, stimulation of CLL cells with phosphorothioate CpG-oligodeoxynucleotide (CpG-ODN) increases their sensitivity to LMB-2 *in vitro* due to upregulation of CD25, an effect also seen to a lesser extent in normal B cells of healthy donors (82). It has not yet been tested in HCL whether TLR9 engagement of HC, or of normal B cells, affects LMB-2 treatment.

Another approach using immunotoxins in HCL is the case of CAT-8015, or Moxetumomab Pasudotox (Moxe), a fusion of the toxin PE to CD22 that showed improved efficacy compared to previous CD22-targeting immunotoxins (83). Clinical benefit was observed in relapsed/refractory HCL patients treated with Moxe in different studies (84, 85). The US Food and Drug Administration (FDA), approved Moxe under the name of Lumoxiti in 2017 for HCL patients after 2 or more prior systemic therapies with at least one being a purine analog. In acute lymphoblastic leukemia (ALL) and in CLL, Moxe showed a limited response rate, probably due to a lower CD22 expression (86–88). Protein kinase C (PKC) activation leads to upregulation of CD22 in CLL (89). In HCL, PKC is constitutively activated, in part due to the interaction of cell adhesion molecules of HC to VN (90), abundantly present in the extracellular matrix of the spleen (**Figure 1E**). This may explain the higher sensitivity to Moxe in HCL, and could imply a different response in key anatomical sites within this disease. Resistance to Moxe in ALL was also linked to alternative splicing of CD22 mRNA and to genome methylation (91, 92), while CD22 antigen downregulation in leukemic cells by monocyte trogocytosis *via* FcγR was described in the context of other anti-CD22 targeted therapies (93). Whether these mechanisms are also ongoing in HCL, remains to be experimentally tested.

The rationale of targeting the BCR signaling represents one of the most successful novel introductions for B-cell neoplasia therapy in the last decade. One example is the Bruton Tyrosine Kinase (BTK) inhibitor ibrutinib, approved for CLL and mantle cell lymphoma (94). In HCL, Sivina and colleagues showed that stimulation of the BCR signaling triggers BTK, ERK and AKT phosphorylation, and that ibrutinib decreases these effects, reducing HC survival (95). Interestingly, ibrutinib also impairs the secretion of CCL3 and CCL4, as well as CXCR4 signaling. These data suggest a possible impact of ibrutinib on the HCL microenvironment interaction at least on three levels: *1)* by affecting BCR signaling induced by microenvironment (auto-)antigens; *2)* by impairing tumor-supporting cell migration mediated by CCL3 and CCL4; and *3)* by redistribution of leukemic cells as consequence of a thwarted CXCR4 cascade (**Figure 1F**). For the moment, ibrutinib monotherapy has been used in single cases of multiple relapse HCLv and in a multicenter trial (NCT01841723), showing clinical benefits (96, 97). Beyond ibrutinib, a second generation BTK inhibitor, acalabrutinib, is currently in one clinical trial for different hematological malignancies including HCL (NCT02362035). The final data of this trial is estimated to be available in two years.

### 2.4 Recent therapeutic options tested in HCL: CAR-T cells and BCL-2 inhibitors

A phase I study of anti-CD22 Chimeric Antigen Receptor-T (CAR-T) cells in patients with relapsed/refractory HCLc and in HCLv is currently being developed (NCT04815356), along with other CAR-T cell approaches (see **Table 1**). The T cell compartment in HCL has been associated to different dysfunctions and linked to a non-responsive state (98). Successful CAR-T therapies highly depend on the extent of immunosuppression within the tumor milieu (99, 100). In HCL, TGF-β1 is present at high levels in BM and PB (101), and this cytokine activates signals that severely hinder T-cell based therapies (102–104). On the other hand, IL-1 actively contributes to the cytokine release syndrome (CRS), which represents one of the main cytotoxic side-effects associated with CAR-T cell therapy (105). Serum levels of IL-1 increase during HCL progression and are elevated as compared to healthy donors and other leukemia and lymphoma patients (106), representing a risky “steady state” scenario that may favor the initiation of CRS (**Figure 1G**). Whether the CAR-T cell approaches will overcome these pitfalls and show patient benefit in HCL is currently an open question of the highest interest.

Venetoclax is a small drug that specifically binds the BH3-binding groove of BCL-2, competing with additional anti-apoptotic members. It is the first BCL-2 antagonist approved for cancer therapy, successfully used in CLL and in acute myeloid leukemia (AML) (107–109). In a recent study, we showed that venetoclax is able to induce cell death in primary HCL samples (25). Importantly, stimulation of T cells through CD3 engagement and co-cultures with HS-5 stromal cells activated primary HC and decreased the pro-apoptotic effect of venetoclax, clearly showing a protective effect of the tumor microenvironment towards BCL-2 inhibition. In addition, stimulation of TLR2 and TLR9, using PAM3 and CpG respectively, also partially rescued the cell death induced by venetoclax (**Figure 1H**). It is currently not known through which mechanisms the activation of HCL cells leads to protection towards BCL-2 inhibition. Venetoclax has been tested, in combination with ibrutinib, in one patient with biclonal IGHV4-34^+^ HCL and CLL, showing promising results (21), but is not currently under any clinical trial for HCL. To our knowledge, no other BH3 mimetics available for hematological malignancies (110) has been tested in HCL until the present.

## 3 Conclusions and perspectives

The TME exerts a protective effect towards some of the most relevant treatment options in HCL, both approved and ongoing experimental molecules, as summarized in **Figure 1**. This opens the path to consider combined therapies to simultaneously attack different TME components and HC.

As examples, interactions between HC and stromal cells could be tackled by different strategies (111), including TGF-β (112) or PKC-β inhibition (113); the latter being already tested in leukemia models using BM stromal cells and showing promising results. Since HC express high levels of CXCR4, interrupting its interaction with CXCL12 by blocking antibody (114), or a drug-mediated inhibition of this axis (115), represent interesting strategies to inhibit the homing of HC to BM. These approaches could be applied in combination with standard treatments directly inducing HC apoptosis. On the other hand, ibrutinib enhances CAR-T cell activity in CLL and in an *in vivo* model of resistant acute lymphocytic leukemia (116), and these therapies are included in different ongoing HCL clinical trials separately (**Table 1**), thus a combined regimen of these two treatments could be considered. Hitherto, the combination of CD20 targeting with cladribine, the targeting of the BRAF-MEK-ERK pathway, CD22 targeting by immunotoxins or CAR-T and BTK inhibition has been developed the furthest towards clinical targeting of the microenvironment in HCL.

In summary, given the vast potential of targetable pathways in the TME landscape of HCL, a new array of therapeutic possibilities remains to be tested in HCL. Due to the rarity of HCL, in addition to testing in clinical trials, any clinical use of such drugs outside trials should also be reported to speed up development of clinical options for patients with HCL. This perspective positions the interactions between HC and their milieu as a key aspect to target in order to increase therapeutic benefit for HCL patients.

## Author contributions

EG and PM wrote the manuscript and created the figure. MG, CN, EM and JP finalized the writing. PM supervised the team. All authors contributed to the article and approved the submitted version.

## Glossary

**Table:**

ADCC	antibody dependent cellular cytotoxicity
AKT	RAC(Rho family)-alpha serine/threonine-protein kinase
ALL	acute lymphoblastic leukemia
AML	acute myeloid leukemia
BCL-2	B-cell lymphoma 2
BCR	B cell receptor
BH3	BCL-2 homology domain 3
BM	bone marrow
BMSCs	bone marrow stromal cells
BRAF	B-Raf proto-oncogene
BTK	Bruton Tyrosine Kinase
CAR-T	Chimeric Antigen Receptor-T
CDA	cladribine
CCL3	Chemokine (C-C motif) ligand 3
CCL4	Chemokine (C-C motif) ligand 4
CCR7	C-C chemokine receptor type 7
CLL	Chronic Lymphocytic Leukemia
CpG-ODN	phosphorothioate CpG-oligodeoxynucleotide
CRS	cytokine release syndrome
CXCL12	C-X-C Motif Chemokine Ligand 12
CXCR4	C-X-C Motif Chemokine Receptor 4
CXCR5	C-X-C chemokine receptor type 5
DCF	pentostatin
ERK	extracellular signal-regulated kinases
FcγR	Fc gamma Receptor
FN	fibronectin
HC	hairy cell leukemia cells
HCL	hairy cell leukemia
HCLc	classic hairy cell leukemia
HCLv	hairy cell leukemia variant
HSCs	hematopoietic stem cells
HSCT	hematopoietic stem cell transplantation
IAP-1	inhibitor of apoptosis protein-1
IFN-α	alpha-interferon
IGHV4-34	immunoglobulin heavy chain gene 34 of family 4
IL	interleukin
M-IGHV	mutated immunoglobulin heavy chain variable region
MAPK	mitogen activated protein kinase
MEK	mitogen-activated protein kinase kinase
NF-κB	nuclear factor kappa-light-chain-enhancer of activated B cells
NK	natural killer (cells)
PAM3	synthetic triacylated lipopeptide
PB	peripheral blood
PE	Pseudomonas exotoxin A
PECAM-1	platelet/endothelial cell adhesion molecule 1
PFS	progression-free survival
PI3Kδ	phosphoinositide 3-kinase delta isoform
PKC	Protein kinase C
sEV	small extracellular vesicles
SYK	spleen tyrosine kinase
TGF-β1	Transforming growth factor beta 1
TME	tumor microenvironment
TNF-α	tumor necrosis factor alpha
TLR	Toll-like receptor
UM-IGHV	unmutated immunoglobulin heavy chain variable region
US	United States
VCAM-1	vascular cell adhesion molecule 1
VLA-4	very late antigen-4
VN	vitronectin
